# Preparation of cellulose nanocrystals from commercial dissolving pulp using an engineered cellulase system

**DOI:** 10.1186/s40643-023-00658-z

**Published:** 2023-07-22

**Authors:** Tiantian Yang, Xuezhi Li, Nuo Xu, Yingjie Guo, Guodong Liu, Jian Zhao

**Affiliations:** 1 State Key Laboratory of Microbial Technology, Shandong University, No.72, Binhai Road, Qingdao, 266237 Shandong China; 2 Henan Province Engineering Laboratory for Bioconversion Technology of Functional Microbes, Henan Normal University, Xinxiang, 453007 Henan China

**Keywords:** *Penicillium oxalicum*, Cellulase system, Cellulose nanocrystals, Enzymatic hydrolysis, Characteristics, Dissolving pulp

## Abstract

There is increasing attention to the production of cellulose nanocrystals (CNCs) from lignocellulosic biomass by enzymatic hydrolysis with cellulase. In this study, the feasibility of the application of a cellulase system from engineered strain *Penicillium oxalicum* cEES in the production of CNCs was assessed. Using commercial eucalyptus dissolving pulp (EDP) as substrate, the CNCs were successfully obtained by enzymatic hydrolysis with the cellulase cEES, and the total yields of CNCs reached 15.7% through three-step enzymatic hydrolysis of total 72 h (24 h for each step). The prepared CNCs were characterized and found that their crystallinity and thermal stability were higher than that of EDP. In the later stage of enzymatic hydrolysis, the process efficiency of enzymatic preparation of CNCs greatly decreased because of the high crystallinity of cellulosic substrate, and a simple homogenization treatment can promote the enzymatic hydrolysis, as well as produce fusiform CNCs with more uniform size and more fermentable sugar that could be further converted into fuels and bulk chemicals through fermentation. This study provides a feasible enzymatic preparation process for CNCs with engineered cellulase and commercial cellulosic materials.

**Graphical Abstract:**

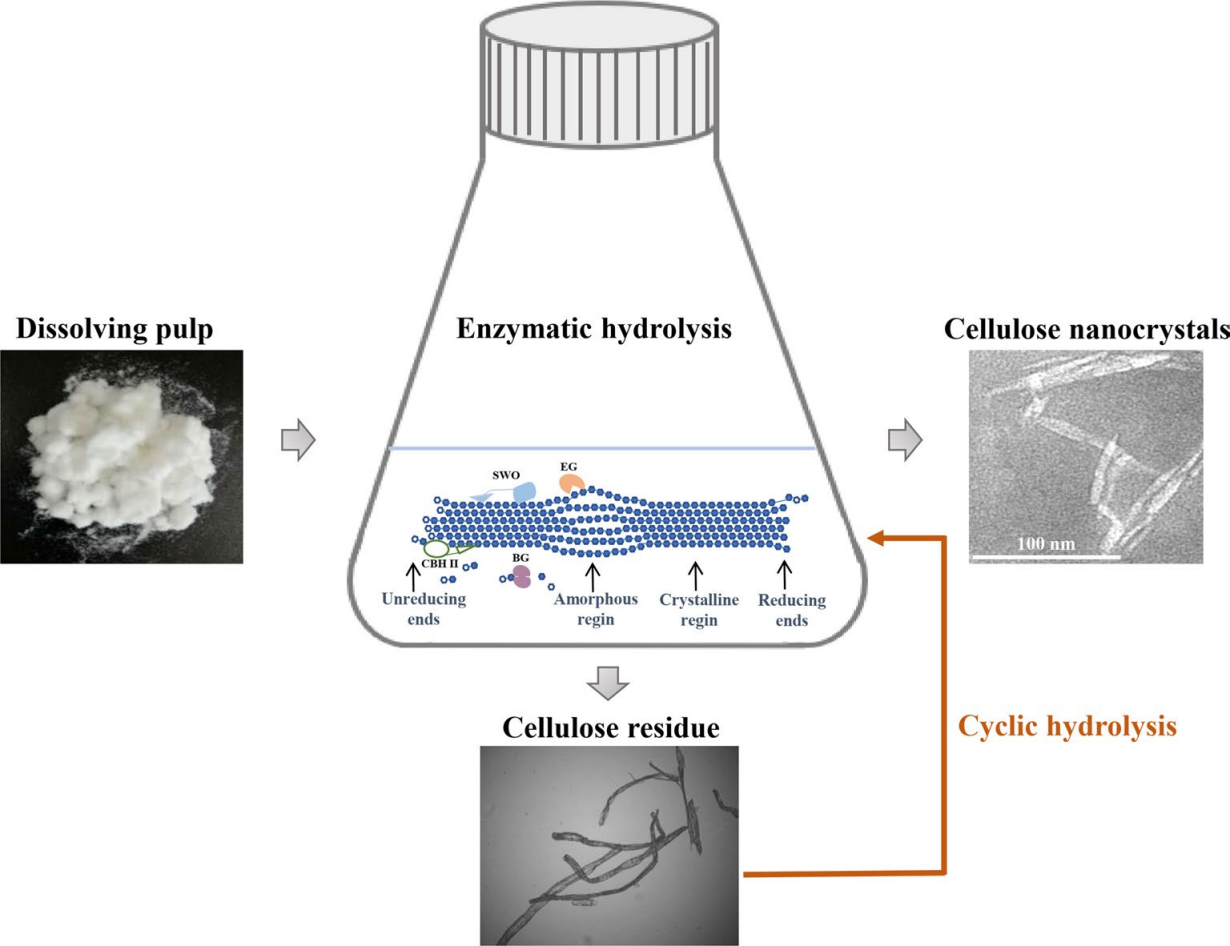

## Introduction

Nanocellulose is a kind of nanomaterial that has attracted much attention because of its unique properties, such as excellent mechanical strength, high surface area, rich hydroxyl groups, biodegradable, high crystallinity and interfacial interaction, etc. (Claro et al. 2019; George et al. 2015; Mondal et al. 2017; Phanthong et al. 2018). It could be extensively applied in the fields of medicine, high-performance materials, packaging (Arantes et al. 2019; Wang et al. 2020; Xu et al. 2019), electrochemical energy storage devices (Guo et al. 2020), viscoelastic functional fluid (Li et al. 2021), water purification (Das et al. 2022), and so on. In general, nanocellulose derived from lignocellulose is divided into cellulose nanocrystal (CNC) and cellulose nanofibers (CNF) (Yang et al. 2020a). CNCs are short (less than 500 nm) and have high crystallinity, whereas CNF contains more amorphous cellulose and high hemicellulose and lignin than CNC (Sharma et al. 2019; Yarbrough et al. 2017).

In general, the CNC was prepared by acid hydrolysis with concentrated acid (Lin et al. 2019; Neto et al. 2016), and the extensively used acids include 64% sulfuric acid, 37% hydrobromic (Shang et al. 2019), 60% phosphoric (Tang et al. 2015), and maleic acid (Bian et al. 2019). In this process, the amorphous cellulose in a cellulosic substrate was first degraded by the concentrated acid under certain conditions, and the crystalline cellulose domain was retained (Lu et al. 2016). The needle-like or rod-like nanocellulose whiskers were obtained by acid hydrolysis. In addition, TEMPO-mediated oxidation can also be used to prepare CNC with negatively charged carboxyl groups (Pacheco et al. 2020; Zhou et al. 2018). However, the chemical processes have some drawbacks, for example, requiring acid-resistant equipment due to the high corrosiveness of acid (Jung et al. 2015), serious acidic wastewater problems, and the high cost of chemicals, such as TEMPO. By contrast, the production of CNCs using enzymatic method is more economically feasible, and environment-friendly because of mild reaction conditions, lower cost, and no waste liquid (Karim et al. 2017). In addition, enzymatic-prepared CNC has rich hydroxyl groups for its functional utilization (Anderson et al. 2014). Up to now, researches in the application of enzymes in CNC preparation mainly included: (i) enzyme was used to pretreat lignocellulosic substrate before mechanical method or acidic hydrolysis, and the enzymes used in the literature included commercial endoglucanase (EG) (An et al. 2016; Dai et al. 2018; Filson et al. 2009; Siqueira et al. 2019); crude cellulase from cultured strains (Yarbrough et al. 2017) and constructed engineered strain (Yang et al. 2020a), and commercial purchase (Aguiar et al. 2020; Pereira et al. 2020). (ii) after chemical or physical treatment, using endoglucanases to hydrolyze lignocellulose to obtain CNCs (Alonso-Lerma et al. 2020; Cui et al. 2016; Teixeira et al. 2015; Xu et al. 2013). In these works, crude cellulase secreted by strains or commercial purchases was usually used, and their unreasonable enzyme system resulted in low process efficiency of enzymatic hydrolysis and low product yield.

It is well-known that cellulase is a complex enzyme system including cellobiohydrolases (CBHs; EC 3.2.1.91 and EC 3.2.1.176), endoglucanases (EGs; EC 3.2.1.4), and β-glucosidase (βG; EC 3.2.1.21) (Lynd et al. 2002). During the enzymatic hydrolysis of cellulosic substrate, endoglucanases randomly act on the internal amorphous region of cellulose to effectively decrease the length of cellulose molecule chains, which play an important role in the enzymatic preparation of CNCs. The presence of a small amount of CBHs also can facilitate this preparation process, because it hydrolyzed glucan chains in the crystalline region of cellulose, thus decreasing the size of substrate particles containing high crystallinity cellulose. In addition, some accessory enzymes, such as hemicellulase (Liu et al. 2013), swollenin (Saloheimo et al. 2002), cellulose induced protein (CIP) (Jia et al. 2021), and so on, can also promote the enzymatic preparation of CNCs by enhancing cellulose degradation and disrupting the plant cell wall. To efficiently produce CNCs by enzymatic hydrolysis, a cellulase system suitable for a substrate is very necessary, but so far, little research about enzyme systems used in the preparation of CNCs is reported.

In our previous works, the effect of four different glycoside hydrolase families EGs from *P. oxalicum* on the preparation of CNCs was evaluated (Yang et al. 2020b), and a genetically engineered strain *P. oxalicum* cEES was constructed for producing the cellulase system containing multi-component proteins. Compared to single EGs from *P. oxalicum,* the engineered cellulase system cEES can hydrolyze microcrystalline cellulose (MCC) more efficiently to produce CNC with more uniform size (Yang et al. 2020a), showing good potential in the enzymatic production of CNCs. Dissolving pulp (DP) is one type of important commercial pulp, and is widely used in the production of regenerated cellulose, rayon, cellophane, cellulose derivatives, such as carboxymethyl cellulose (CMC), methylcellulose (MC), hydroxypropyl cellulose (HPC), etc. (Cao et al. 2014; Kumar et al. 2017). DP is mainly composed of cellulose (> 90%, on the dry weight of pulp), thus it is expected to be a good raw material for preparing CNC because of its lower cost than MCC. In this study, we investigated the feasibility of the engineered cellulase system cEES in application in CNC preparation from DP through the step hydrolysis process and continuous hydrolysis process, respectively. The prepared CNCs were characterized by transmission electron microscope (TEM), X-ray diffraction (XRD), Thermogravimetric (TG) analysis, and Fourier transform infrared spectroscopy (FT-IR).

## Materials and methods

### Materials and strains

*P. oxalicum* cEES was constructed by overexpressing the genes *cel7B* and *cel5B* from *P. oxalicum* and *swollenin1* from *Trichoderma reesei*, and knocking out *cbh1* in parent strain *P. oxalicum* M12 according to (Yang et al. 2020a), and preserved in our laboratory. EDP was purchased commercial pulp.

### Enzyme production

The spores of the engineered strain cEES were cultured in glucose media (2% glucose, 50 × vogel’s salt) at 30 °C and 200 rpm for 1 day, then the mycelia obtained by filtration were transferred to the fermentation medium (0.2% (NH_4_)_2_SO_4_, 0.28% NaNO_3_, 0.1% urea, 0.3% KH_2_PO_4_, 0.05% MgSO_4_, 2% corn residue, 0.6% microcrystalline cellulose, 4.66% wheat bran and 1.0% soybean cake powder) with a dosage of 0.5 g mycelia per 100 mL in 500 mL shake flasks after culture of 6 days, the fermentation liquid was filtrated and centrifuged, and the supernatant was used as cellulase solution for hydrolysis of EDP.

### Analytical methods

The concentration of protein in the cEES enzyme solution was measured by the Bradford method (Yang et al. 2020a). The supernatant was added into Bradford and mixed evenly reacting for 5 min. Then, the absorbance of the solution was measured at 595 nm and the protein concentration of the supernatant was calculated according to the standard curve prepared with 1 mg/mL BSA.

1% xylan, 1% carboxymethylcellulose sodium (CMC-Na) solution (Sigma, USA), and Whatman No. 1 filter paper (Shanghai, China) were, respectively, used as the substrate for assaying the activities of xylanase, EG, and FPA (Li et al. 2015). The diluted enzyme solution (0.5 mL) was added to the substrate (1.5 mL) reacting at 50 °C for 30 min, then 3 mL of DNS was added to terminate the reaction. Before distilled water (20 mL) was added, the reaction system was boiled for 10 min, then the OD of the solution was measured at 540 nm.

0.1% *p*NPC (Sigma, USA) solution composed of 0.1% D-gluconolactone (Sangon Biotech) was used as a substrate for assaying the activities of CBH. The diluted enzyme solution (0.1 mL) was added to the substrate (0.05 mL) reacting at 50 °C for 30 min, then 0.15 mL of 10% Na_2_CO_3_ was added to terminate the reaction. Then, the OD of the solution was measured at 420 nm.

Chemical components of EDP, including the contents of cellulose, xylan, and lignin, were determined according to the relevant methods published by the National Renewable Energy Laboratory (NREL) (Sluiter et al. 2012). Briefly, 0.3 g dry weight of the pulp was first extracted with ethanol for 12 h and dried at room temperature. The extracted pulp was hydrolyzed with 3 mL of 72% sulfuric acid for 1 h at 30 °C, and acid concentration was adjusted to 4% with 84 mL of distilled water and further hydrolyzed at 121 °C for 1 h. After the two-step acid hydrolysis, the sample was filtrated, and the solid was dried at 105 °C until constant weight. To determine acid-soluble lignin content, the partly separated liquid was diluted with 4% sulfuric acid to a suitable concentration, and absorbance at 240 nm was measured using an ultraviolet spectrophotometer (UV-2550, Shimadzu Co.). The contents of acid-soluble lignin and acid-insoluble lignin were calculated according to formulas (1) and (2), respectively. Total lignin content was the sum of acid-insoluble lignin and acid-soluble lignin:

1 $$\mathrm{acid}-\mathrm{insoluble lignin }(\mathrm{\%})=\frac{\mathrm{M}1}{\mathrm{M}}\times 100\%$$

2 $$\mathrm{acid}-\mathrm{soluble lignin }(\mathrm{\%})=\frac{{OD}_{240}\times 0.087\times n}{25\times \mathrm{M}\times \mathrm{P}}\times 100\mathrm{\%}$$

where M was the dry weight of extracted pulp sample (g), M1 was the dry weight of acid-insoluble lignin (g), n was dilution multiple, 0.087 was the volume of filtrate (L), 25 was the absorptivity of the samples at 240 nm (L/g.cm^−1^), P was pathlength of UV–Vis cell (cm).

3 mL of the above-separated liquid was neutralized with Ba(OH)_2_ to adjust pH to neutral, then filtered with 0.22 μM aqueous membrane, and the concentrations of sugar components in the supernatant were detected by HPLC with Hpx-87p chromatographic column (Bio-Rad). The column temperature was set at 78 °C, the purified water was used as the mobile phase, the flow rate was 0.5 mL/min, and a differential refractive index detector was used.

The contents of cellulose and xylan were calculated according to formulas (3) and (4), respectively:

3 $$\mathrm{cellulose content} (\%)=\frac{{\mathrm{C}}_{1}\times \mathrm{V}}{\mathrm{M}}\times 0.9\times 100\mathrm{\%}$$

4 $$\mathrm{xylan content }(\mathrm{\%})=\frac{{\mathrm{C}}_{2}\times \mathrm{V}}{\mathrm{M}}\times 0.88\times 100\mathrm{\%}$$

where C_1_ and C_2_ were the concentrations of glucose and xylose, respectively (mg/mL), V was the volume of the reaction system (mL), and M was the weight of the pulp (mg). All experiments were triplicate, and the mean value was shown in the paper.

### Preparation of CNCs

The pulp board of EDP was cut into strips of about 1 × 2 cm, then dispersed by beating with a disintegrator (30 s, 1 kW) and ground with a ball mill (FRITSCH, PULVERISETTE 5/4, Germany) for 180 min at 150 rpm. The treated pulp was used as a substrate for preparing CNCs.

**Fig. 1:**
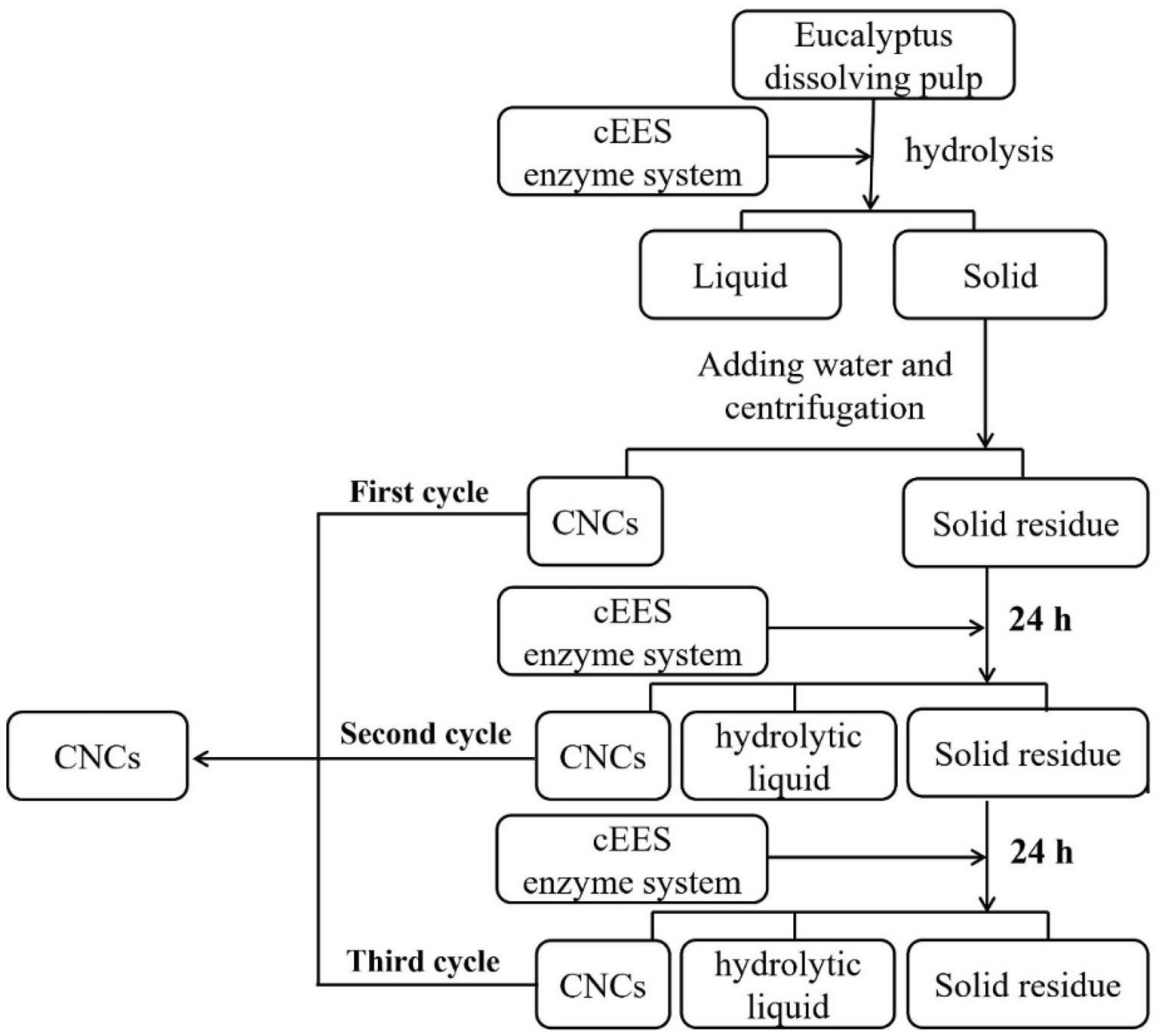
Schematic diagram of the preparation of CNCs through step enzymatic hydrolysis with the engineered cellulase system

For preparing CNCs, the EDP of 0.15 g (dry weight) was dispersed in 4 mL of NaAc-HAc buffer (0.1 M, pH 4.8). Then, the cEES enzyme and buffer were added to conduct enzymatic hydrolysis in a 50 mL flask with a working volume of 15 mL under the conditions of 1% pulp concentration, enzyme dosages of 30 mg protein per gram glucan, 50 °C, 150 rpm according to the flow chart (Fig. 1). After 24 h of hydrolysis, the enzyme was inactivated by boiling for 10 min. The hydrolysate separated by filtration was analyzed by HPLC for calculating the yield of glucose. The separated unconverted cellulose was re-suspended with distilled water of 45 mL, centrifuged at 1600 g for 10 min, and the upper suspension containing CNCs was collected (Yang et al. 2020a). The remaining unconverted residue was used for the second and third cycles of hydrolysis using the same procedure as the first cycle. Besides, the 48 h continuous hydrolysis was carried out to separate the CNCs directly. The total yields of CNCs were calculated according to formula (5).

5 $${\mathrm{Y}}_{\mathrm{N}}(\mathrm{\%})=\frac{{\mathrm{M}}_{2}}{{\mathrm{M}}_{1}}\times 100\mathrm{\%}$$

where Y_N_ was the yield of CNCs (%), M_1_ was the quality of EDP in the reaction system (g), and M_2_ was the quality of dried CNCs obtained (g).

All experiments were in triplicate. The average was calculated as the final value in the paper, and error bars represent the standard deviations.

The yield of glucose was calculated according to the formula (6).

6 $${\mathrm{Y}}_{G}(\mathrm{\%})=\frac{\mathrm{C}\times \mathrm{V}}{\mathrm{M}\times \mathrm{P}}\times 0.9\times 100\mathrm{\%}$$

where Y_G_ was the yield of glucose (%), C was the concentration of glucose (mg/mL), V was the volume of the reaction system (mL), M was the dry weight of EDP for enzymatic hydrolysis in the system (mg), P was the proportion of glucan in EDP (%), 0.9 was the conversion factor from glucose to glucan.

For mechanical treatment-assisted enzymatic hydrolysis, the solid residue from the first-step enzymatic hydrolysis of EDP (for 24 h) was used as substrate. Before the second enzymatic hydrolysis, the solid residue was first treated by a homogenizer (IKA T18, Germany) under different power and time, then separated by filtering, and hydrolyzed with the cellulase cEES of 30 mg protein/g substrate (on the dry weight of substrate). The CNCs were obtained according to the method mentioned above, and the glucose concentration in hydrolysates was analyzed by HPLC to calculate the glucose yield.

### Characterization of CNCs and residue

#### Particle size distribution and Zeta potential values

The particle size distribution and Zeta potential values of the CNC suspensions were determined by the Zeta Plus (Brookhaven, America) analyzer.

#### FT-IR

Infrared spectra were measured with diffuse reflectance (NICOLET iS10, America) between 4000 and 400 cm^−1^, with a resolution of 4 cm^−1^ and 16 times of scans. The dried sample was mixed with KBr powder using an agate mortar.

#### TEM observation

TEM image was used to observe the morphology and size of prepared CNCs. The sample suspensions were drop-cast on a carbon mesh, air-dried overnight, stained with phosphotungstic acid, and observed by electron transmission microscopy (FEI Tecnai G2 F20).

#### XRD analysis

XRD patterns of the dried samples were determined using the X-ray diffractometer (Ultima IV, Japan). The XRD uses CuKα radiation generated at 40 kV and 40 mA. Scans were carried out in a 2θ range from an angle of 5° to 50° at a scanning rate of 5°/min. The baselines in XRD patterns were first levelled with origin 9.1 software to remove environmental background, and the crystallinity index (CrI) was calculated from the XRD patterns according to formula (7) (Yao et al. 2021):

7 $$\mathrm{CrI}=\frac{{I}_{200}-{I}_{\mathrm{am}}}{{I}_{200}}\times 100\%$$

where* I*_200_ was the maximum peak intensity at 2θ between 22° and 23°, and *I*_am_ was the minimum diffraction intensity at 2θ between 18° and 19°.

#### TG analysis

The thermal stability of dried samples was analyzed using a thermogravimetric analyzer (TG209F3 Tarsus, Germany). The dried samples (10–20 mg) were heated from 30 to 900 °C at a rate of 10 °C/min in a nitrogen atmosphere (Jia et al. 2017).

## Results and discussion

### Chemical compositions of EDP and characteristics of the engineered cellulase system from engineered strain cEES

Table 1 presents the main chemical compositions of EDP. The EDP contains 91.75% cellulose, 2.42% hemicellulose, and 0.74% lignin. The very low contents of hemicellulose and lignin should be attribute to the preparation method of dissolving pulp (Dong et al. 2020). The high purity of cellulose in EDP was very beneficial for the preparation of CNCs by enzymatic hydrolysis with cellulase. The slight hemicellulose content means that xylanase was also needed for the enzymatic hydrolysis of EDP.

**Table 1:** Chemical compositions of Eucalyptus dissolving pulp (%, on dry pulp weight)

Cellulose	Xylan	Lignin	Others
91.75 ± 0.13	2.42 ± 0.04	0.74 ± 0.03	5.08 ± 0.13

Table 2 displays the different enzymatic activities of the cellulase system from engineered strain cEES and parent strain *P. oxalicum* M12, respectively. It was found that, compared to the cellulase system from strain M12, the engineered cellulase system from strain cEES had higher enzymatic activities of EG, CBH, and FPase, a higher ratio of the activity of EG/CBH (51.46 for cEES *vs* 36.24 for M12, respectively) and EG/FPase (57.40 for cEES *vs* 50.51 for M12, respectively).

**Table 2:** Enzymatic activities (U/mL) of cellulases from engineered strain *P. oxalicum* cEES and parent strain *P. oxalicum* M12, respectively

Strains	EG	CBH	EG/CBH	FPase	EG/FPase	xylanase
cEES	29.85 ± 0.15	0.58 ± 0.01	51.46	0.52 ± 0.05	57.40	44.90 ± 0.16
M12	16.67 ± 0.10	0.46 ± 0.01	36.24	0.33 ± 0.04	50.51	–

– not measured

The high enzymatic activities of the engineered cellulase system cEES mean that less enzyme dosage than cellulase from strain M12 was required in the enzymatic hydrolysis of EDP, decreasing enzyme cost for producing CNC. The high activity ratios of EG/CBH and EG/FPase should be attributed to overexpressing of two EG genes (*cel7B* and *cel5B*) from *P. oxalicum,* and knocking out of gene *cbh1* in parent strain *P. oxalicum* M12. It was beneficial to the enzymatic preparation of CNCs, because the relatively more EG in the engineered cellulase system can make the amorphous region of cellulose degrade rapidly, resulting in effectively decrease in the length of cellulose molecule chains, as well as a small amount of CBH hydrolyzed glucan chains in the crystalline region of cellulose to decrease the size of substrate particle containing high crystallinity cellulose to the nanoparticle. FPase could hydrolyze soluble cellulose oligosaccharides such as cellobiose into glucose for decreasing oligosaccharides inhibition on CBH and EG. Besides, *swollenin1* from *T.reesei* was simultaneously overexpressed in the engineered cellulase system cEES, which can partly destroy the crystalline structure of cellulose, enhancing the enzymatic hydrolysis process of EDP with EGs. As some xylan existed in the cellulosic substrate (Table 1), the xylanase in the cellulase system cEES can disrupt the structure of the cell wall by removing the xylan wrapped on the surface of cellulose, thus enhancing enzymatic preparation of CNCs. Based on the above results analysis, it was deduced that the engineered cellulase system cEES containing multi-component proteins have the potential for producing CNCs from EDP through the enzymatic hydrolysis method.

### Enzymatic preparation of CNCs using cEES cellulase system

**Fig. 2:**
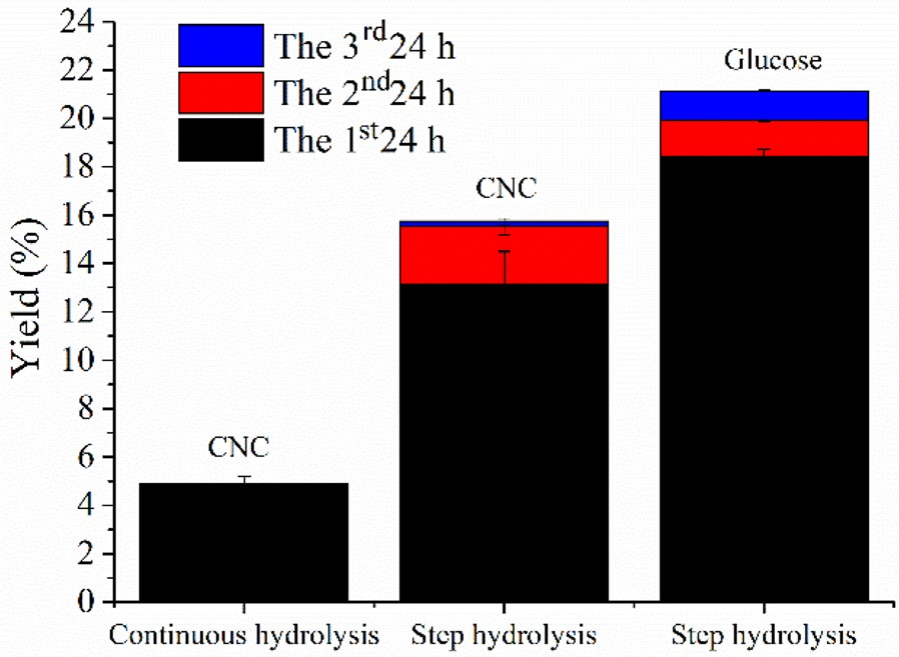
Yields of CNCs and glucose produced by step hydrolysis for 72 h (each step for 24 h) and continuous hydrolysis for 48 h with the engineered cellulase cEES of 30 g protein per gram pulp

Based on preliminary experiments, the engineered cEES cellulase of 30 mg protein per gram substrate (dry weight) was used to hydrolyze EDP for preparing CNCs through step hydrolysis process and continuous hydrolysis process, respectively. The experimental results are shown in Fig. 2.

Figure 2 shows the cumulative CNC yield reached 15.7% by three steps of hydrolysis, and the first step (first-24 h) contributed the most to the CNC yield, while the second and third hydrolysis stages decreased in turn. The third stage (third-24 h) had the lowest CNC yield (just 0.2%). The change in yield of glucose obtained by the step process was similar to the change in CNC yield, and the smallest amount of glucose was obtained in the third step of enzymatic hydrolysis. This phenomenon may be explained as follows, in the early stage of enzymatic hydrolysis, there was a high proportion of amorphous region in the cellulose of EDP, which can be rapidly degraded by EGs in the engineered cellulase system to form nanoparticles and soluble oligosaccharides, then further degraded into glucose because of existing of FPase activity in the engineered cellulase cEES. However, in the later stage of enzymatic hydrolysis, the amount of cellulose sited in the crystalline region increased, resulting in low degradation efficiency, because EG was the main component in the engineered cellulase cEES. Yoshida et al. (2008) also observed that the amorphous cellulose was preferentially hydrolyzed than crystalline cellulose.

It was also found in Fig. 2 that the yield of CNCs prepared from continuous enzymatic hydrolysis of 48 h (4.9%) was much lower than that from the step-hydrolysis process of total 48 h (24 h + 24 h, total yield of 15.5%) at same cellulase dosage, possibly because some small particles of CNCs were further degraded into soluble sugar during the continuous hydrolysis, but in the step hydrolysis process, the CNC particles produced from the first step of enzymatic hydrolysis were separated out, avoiding further being degraded in subsequent enzymatic hydrolysis. the results indicate the step hydrolysis process was more suitable for producing CNCs through enzymatic hydrolysis with the engineered cellulase system. Further work will focus on the optimization of enzymatic hydrolysis conditions and further modification of the engineered cellulase system to increase the yield of CNCs prepared by enzymatic hydrolysis.

### Characterization of CNCs prepared by enzymatic hydrolysis of EDP

#### Crystallinity

Using XRD analysis, the crystallinity of CNC samples prepared by different enzymatic hydrolysis of EDP with the engineered cellulase cEES was determined and found that the crystallinity degrees of EDP, the CNC from the first step enzymatic hydrolysis (first 24 h) and the second step hydrolysis (the second 24 h), respectively, and CNC from continuous enzymatic hydrolysis of 48 h were 69.3%, 87.3%, 92.7%, and 93.4%, respectively. The higher crystallinity degree for all the CNC samples than EDP was attributed to the degradation of cellulose in the amorphous region in EDP. It was also found that the difference in crystallinity between EDP and CNC from 1st step hydrolysis of 24 h was higher than that between 1st step hydrolysis and 2nd step hydrolysis (24 h for each step), which should be due to the amorphous cellulose of EDP rapidly being hydrolyzed in the first 24 h, resulting in the rapid increase in crystallinity of CNCs in the early hydrolysis stage. The higher the crystallinity of the substrate, the more the restriction of enzymatic hydrolysis (Hall et al. 2010). Therefore, compared to the EDP, the solid residue from the first step of enzymatic hydrolysis was more difficult to degrade by the engineered cellulase because of its low CBH activity. In addition, xylanase in the engineered cellulase system hydrolyzed hemicellulose in EDP, which also partly contributed to the crystallinity of CNCs increase, because hemicellulose is an amorphous substance.

#### FT-IR analysis

**Fig. 3:**
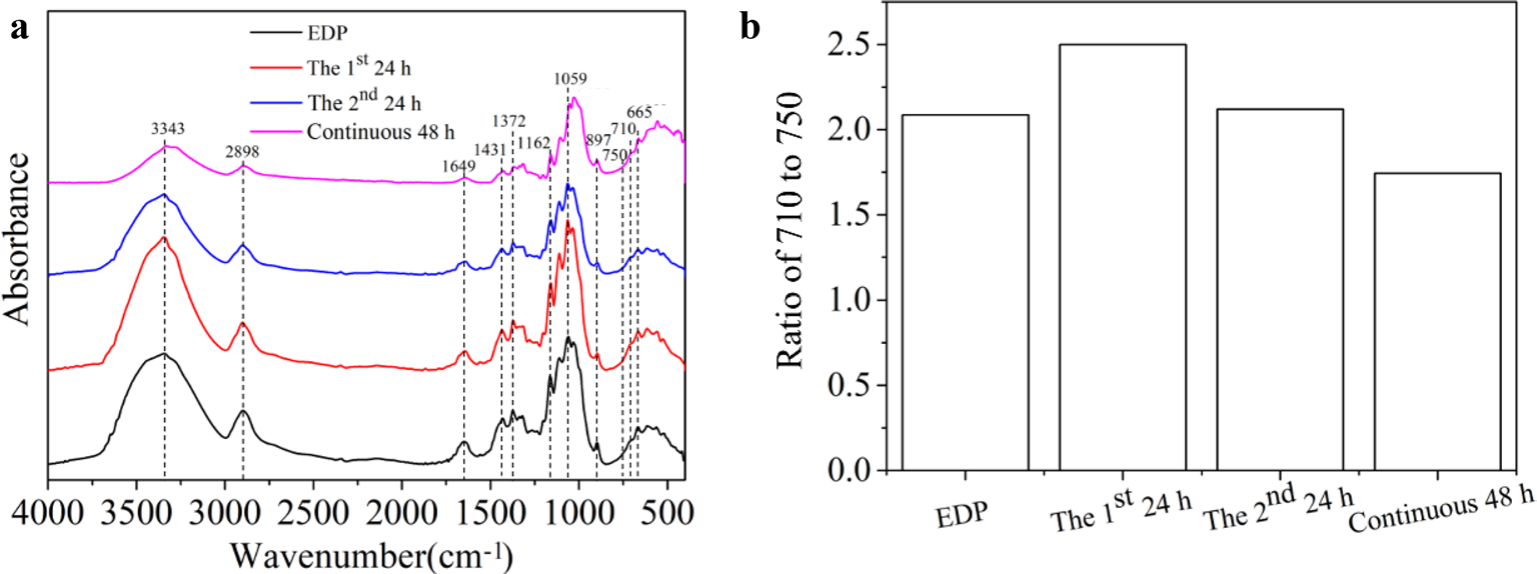
FTIR spectra (**a**) and the ratios of absorbances at 710 cm^−1^ and 750 cm^−1^ (**b**) of EDP and the CNCs prepared by different enzymatic hydrolysis with the cEES cellulase system

Figure 3 shows the FTIR spectrum of EDP and the CNCs prepared by enzymatic hydrolysis with the engineered cEES cellulase system. As shown in Fig. 3a, there was only one peak at 4000–2995 cm^−1^ standing for the OH stretching vibration of the hydrogen bond, and its absorption strength for CNCs prepared from 1st 24 h of enzymatic hydrolysis was significantly enhanced compared with EDP, indicating that the hydrogen bonds inside the material were broken in the enzymatic hydrolysis. The absorbance at 2900 cm^−1^ represents the stretching vibration of the C–H (Seta et al. 2020), and the absorption peak of 1645 cm^−1^ represents the O–H deformation, which might cause by cellulose and water (Macedo et al. 2020). The absorbance at 897 cm^−1^ represents the amorphous region in cellulose (Hospodarova et al. 2018; Poletto et al. 2014), and was a characteristic of β-glycosidic bond vibration (Liu et al. 2019). The absorption peak of Iβ-type cellulose was at 710 cm^−1^, and Iα-type cellulose was at 750 cm^−1^ (Gu et al. 2013; Sugiyama et al. 1991), and the ratio of their absorbance values can partly display the changes of different types of cellulose during the enzymatic hydrolysis. It is shown in Fig. 3b that the value of A710/A750 of CNCs prepared by the enzymatic hydrolysis of 24 h and 48 h (24 h + 24 h), respectively, were higher than that of EDP, indicating the more Iα-type cellulose being degraded during enzymatic hydrolysis of EDP with the engineered cellulase system. It has been reported that the Iα-type cellulose is the triclinic crystal structure, and is easier to degrade than the Iβ-type cellulose of monoclinic crystal (Carlsson et al. 2015). However, the value of A710/A750 of the CNCs prepared by continuous hydrolysis of 48 h was lower than that of EDP and the CNCs from the step hydrolysis process, which may be related to the crystalline region containing more Iβ-type cellulose being destroyed by expressed Swollenin in the engineered strain cEES.

#### Zeta potential values

The zeta potential (ζ) of CNC suspensions produced from step enzymatic hydrolysis of EDP with the engineered cellulase cEES was measured and found that the zeta potential values of CNC obtained from the first and the second 24 h enzymatic hydrolysis were − 17.46 ± 2.64 mV and − 18.61 ± 2.36 mV, respectively, indicating poor stability for the CNC suspensions. Typically, zeta potential greater than ± 30 mV is regarded as stable. Aguiar et al. (2020) obtained the CNCs suspension with zeta potential values of approximately − 25 mV from cotton bagasse and straw by enzymatic hydrolysis.

#### Morphology

**Fig. 4:**
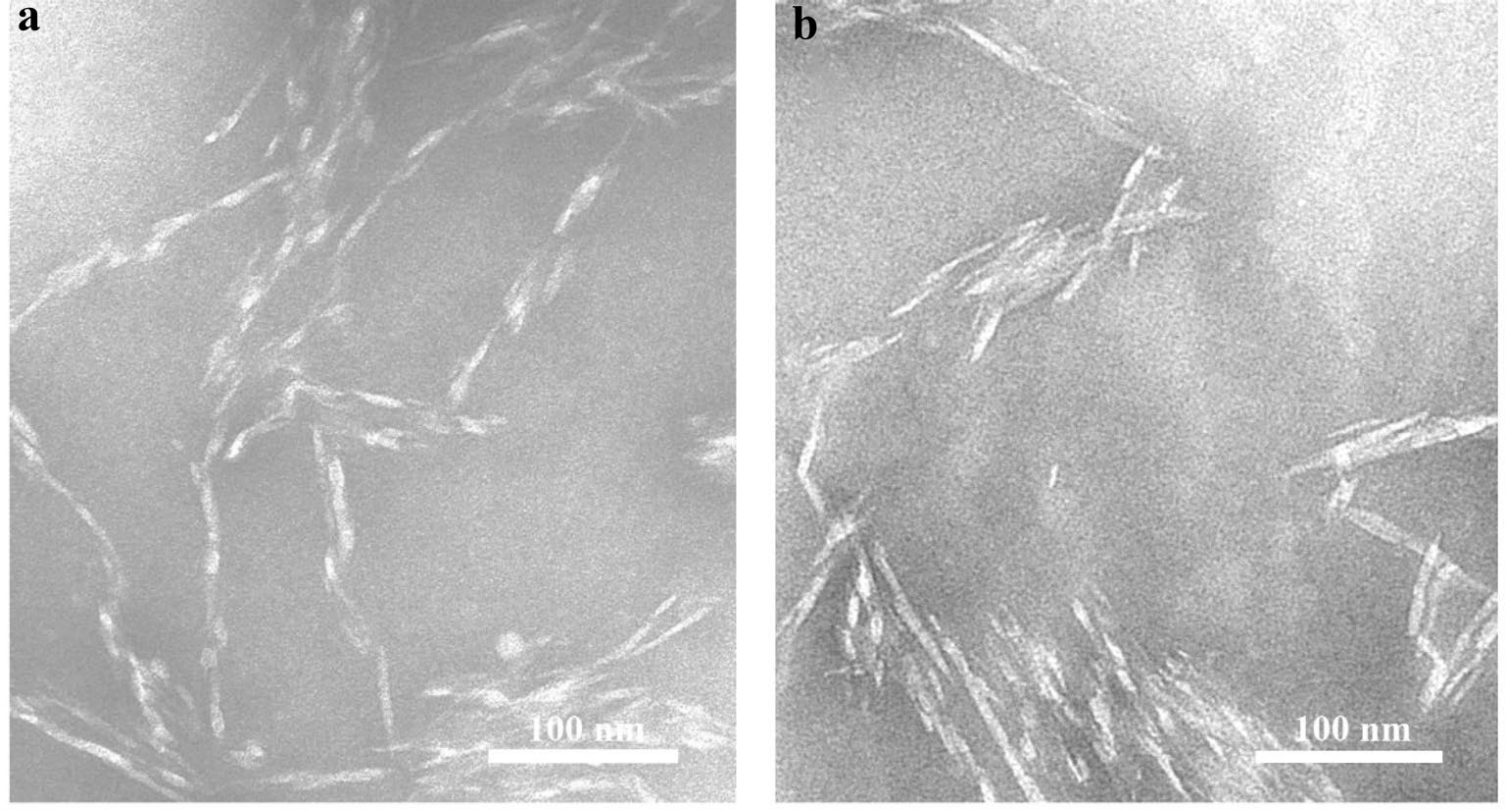
TEM images of CNCs produced by enzymatic hydrolysis of 1st 24 h (**a**) and 2nd 24 h (**b**) with the engineered cellulase system

It was observed by TEM as shown in Fig. 4, that the CNCs prepared by step enzymatic hydrolysis of EDP with the engineered cellulase cEES were fusiform-shaped particles, which was different from the needle-like or rod-like nanocellulose whiskers obtained by sulfuric acidic hydrolysis. It has been reported that the morphology of CNCs was depend on their source and preparation methods (Chang et al. 2019).

### Characteristics of solid residue after enzymatic hydrolysis

#### XRD analysis

**Fig. 5:**
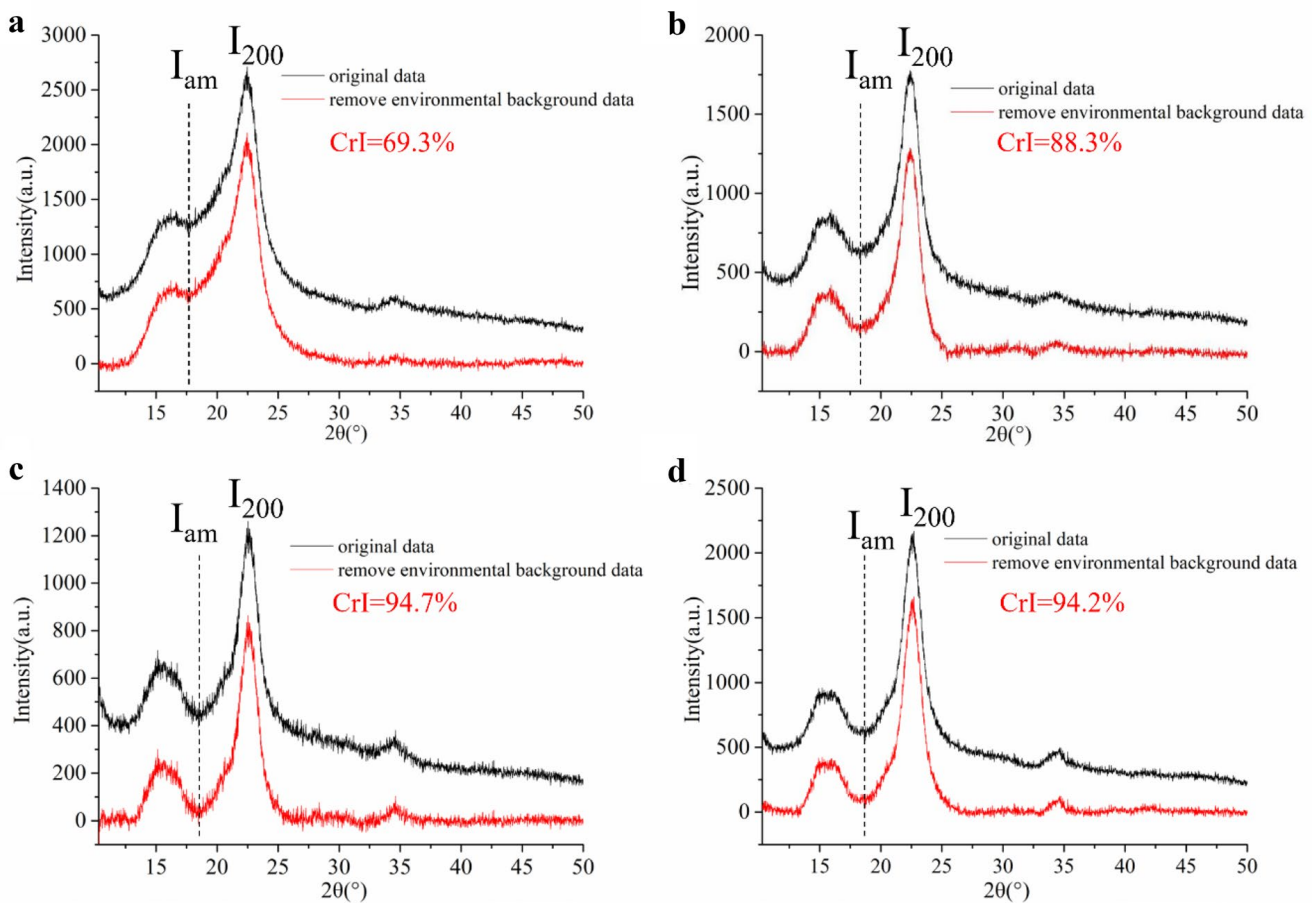
XRD patterns and crystallinity of EDP (**a**) and solid residues from the 1st 24 h hydrolysis (**b**), the 2nd 24 h hydrolysis (**c**), and continuous hydrolysis of 48 h (**d**), respectively

XRD patterns and crystallinity of the solid residues from different enzymatic hydrolysis processes are shown in Fig. 5. It was found that the XRD patterns of all samples existed typical peaks at 2θ = 16.4°, 22.4°, and 34.5°, which belonged to crystalline type I cellulose structure (French et al. 2013; Sun et al. 2015), indicating that enzymatic hydrolysis did not affect the crystal structure type of cellulose. The crystallinity of all the solid residues from enzymatic hydrolysis was higher than that of EDP, which should be attributed to the degradation of cellulose in the amorphous region. Abundant EGs in the engineered cEES enzyme system, as shown in Table 2, enable it to effectively hydrolyze the cellulose in the amorphous region of EDP, but deficient CBH limits the hydrolysis of crystalline cellulose. It also explains why the yield of CNCs obtained from the first step of enzymatic hydrolysis was higher than that from the second step of enzymatic hydrolysis.

It was also found that the solid residue from continuous enzymatic hydrolysis of EDP for 48 h had very high crystallinity (94.2%), indicating that most of the cellulose sited in the amorphous region had been hydrolyzed. From this, it can also be inferred that even if the hydrolysis time was extended, it was difficult to effectively increase the yield of CNC because of the deficient CBH in the engineered cellulase system. From Fig. 2, it was known that the yield of CNC obtained by continuous enzymatic hydrolysis of EDP was low; therefore, it was concluded that the step hydrolysis process was more suitable for preparing CNCs through enzymatic hydrolysis with the engineered cellulase cEES.

#### TG analysis

**Fig. 6:**
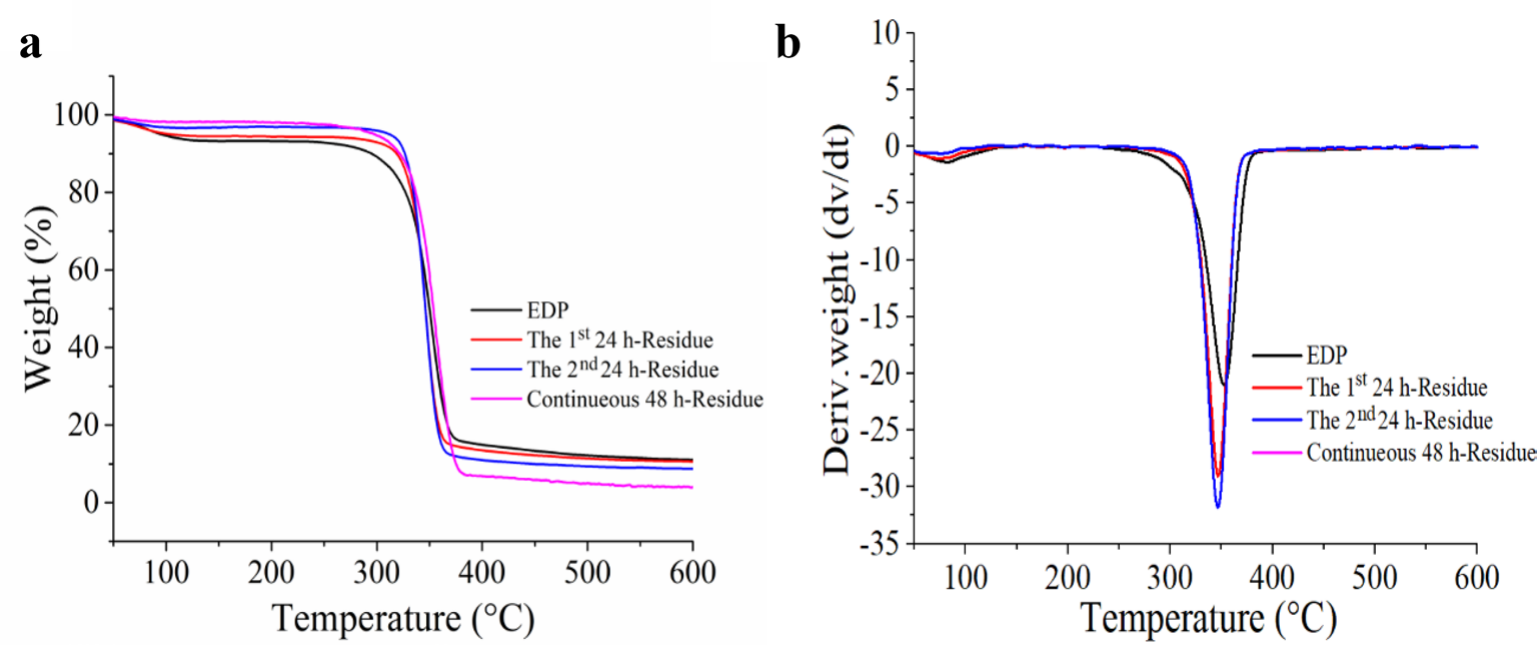
Thermal stability analysis of EDP and solid residues separated from different enzymatic hydrolysis processes. In which, (**a**) TG curve; (**b**) DTG curve

Table 3 shows the initial onset temperature (T_onset_) and maximal weight loss temperature (T_max_) of EDP and solid residues from different enzymatic hydrolysis processes. Figure 6 displays the curves of TG and DTG. As shown in Fig. 6, the weight loss between 40 ° and 150 °C was due to the loss of absorbed water in the materials, including adsorbed water and intermolecular hydrogen bond water (Li et al. 2011). Hemicellulose decomposition occurred in the region of 220 °C to 300 °C, and it was found that, in this region, the mass change of solid residues was lower than that of EDP, which was consistent with the results in the literature (Abraham et al. 2013; Manfredi et al. 2006; Saelee et al. 2016). This might be attributed to the hemicellulose degradation during enzymatic hydrolysis, because the engineered cellulase system contained xylanase.

**Table 3:** T_onset_ and T_max_ of EDP and solid residue separated from the different enzymatic hydrolysis process

	EDP	The 1st 24 h-Residue	The 2nd 24 h-Residue	Continuous 48 h-Residue
T_onset_ (°C)	235.4	278.4	285.4	230.5
T_max_ (°C)	352.5	346.7	346.5	356.1

Table 3 shows that the thermal degradation of EDP in the N_2_ atmosphere started at 235.4 °C, and it was increased to 278.4 °C and 285.4 °C, respectively, for the solid residues from enzymatic hydrolysis of 24 h and 48 h (two-step, 24 h for each step) with the cEES cellulase system, indicating that the thermal stability of the solid residues increased after enzymatic hydrolysis. It could be related to the differences in the proportion of crystalline cellulose in different samples. In general, the higher the crystallinity, the better the thermal stability. However, compared to the solid residues from the step hydrolysis process, the initial onset temperature (230.5 °C) of the solid residue obtained by continuous hydrolysis of 48 h was lower, but the maximal weight loss temperature (T_max_) was higher, which may be related to other factors such as hemicellulose content, the length of cellulose chains except for the cellulose crystallinity.

### Preliminary study on the feasibility of mechanical treatment on improving enzymatic hydrolysis of solid residues

**Fig. 7:**
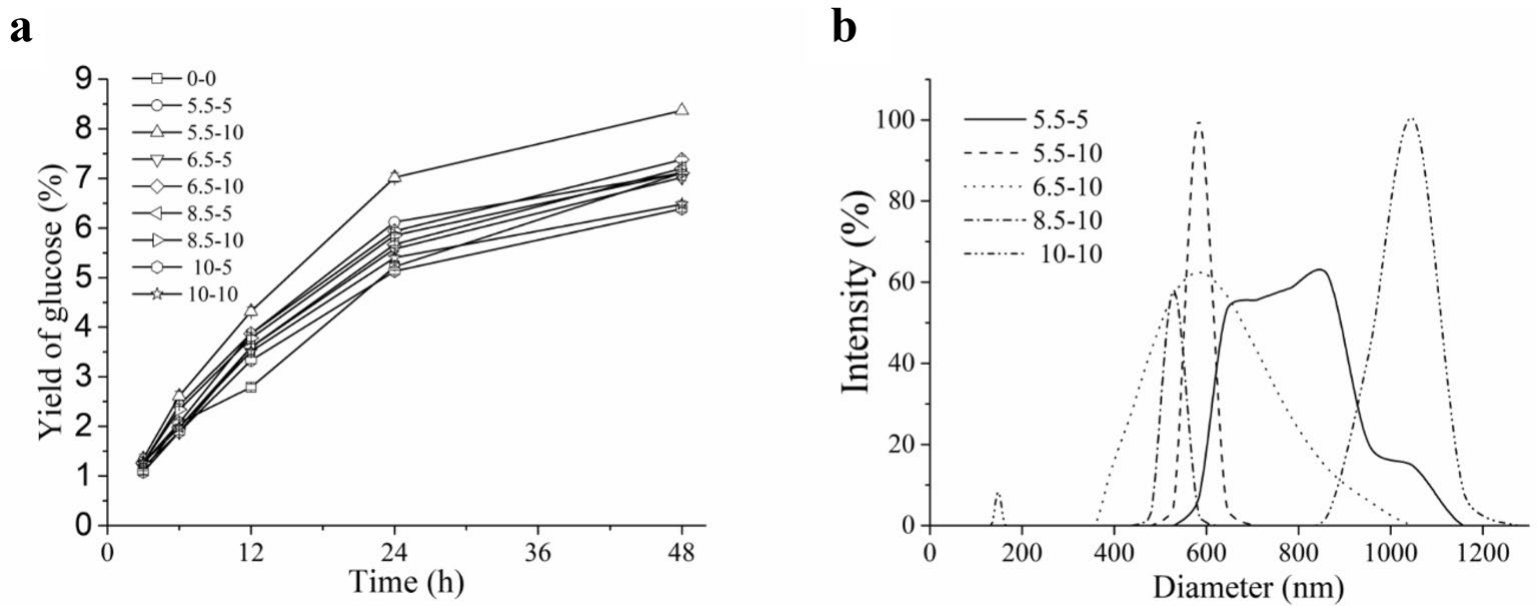
Yield of glucose (**a**) and particle size distribution of CNCs (**b**) obtained by mechanical treatment-assisted enzymatic hydrolysis of the solid residue using the cEES enzyme system

From the above research, it can be seen that the hydrolysis efficiency of cellulosic substrate became very low in the later stage of enzymatic hydrolysis (Fig. 2) because of high crystallinity (Fig. 5). To increase the accessibility of enzymes and improve the efficiency of enzymatic hydrolysis, in this study, the effect of a simple mechanical treatment on the enzymatic hydrolysis of the high-crystallinity solid residue obtained by enzymatic hydrolysis of 24 h was assessed. In the process, the solid residue was first treated with a homogenizer under different strengths and different times, then was hydrolyzed with the engineered cellulase system from strain cEES for 24 h. It is found from Fig. 7a that a simple mechanical treatment can promote the enzymatic hydrolysis of solid residue, and increase the yield of glucose in the enzymatic hydrolysis. The highest glucose yield of 7.1% was obtained by mechanical treatment under the condition of homogenizer power of 5.5 kW for 10 min (Fig. 7a), 1.9% higher than that of the control (without mechanical treatment). On the other hand, the particle size of the obtained CNCs became smaller, and the size distribution became more uniform (only one peak in Fig. 7b) through the simple mechanical treatment.

Table 4 presents a comparison of the process used for preparing CNCs in this study and the traditional processes reported in the literature. Using the sulfuric acid hydrolysis process, rod-shaped CNCs with high yield could be obtained, but it required acid corrosion resistant equipment, as well as produced a great quantity of acid waste. TEMPO oxidation method is also usually used for preparing CNCs, but its disadvantages included the high cost of chemicals, requiring corrosion resistance equipment due to the usage of oxidants and alkali, producing alkaline wastewater containing TEMPO. Pretreatment with cellulase or endoglucanase prior to a chemical method can reduce chemical dosages in subsequent chemical treatment but also resulted in challenges similar to conventional chemical methods. Enzymatic preparation of CNCs with only enzymes without chemical treatment had some advantages including mild reaction conditions, environmentally friendly, no need for corrosion-resistant equipment, producing sugars liquid that could be further converted into high-value products by fermentation, and so on. However, the current disadvantages of this process are low CNC yield and long treatment time because of lacking in efficient enzyme preparations specifically designed for CNC production. Compared to the results obtained by enzymatic hydrolysis with the only enzyme in literature (Table 4), this study preliminarily proved the potential of the engineered cellulase system cEES in CNC production. It is expected to further increase yield by optimizing the pretreatment of cellulosic feedstock for destroying the crystalline structure of cellulose, optimizing enzymatic hydrolysis process and reaction conditions, and further improving the cEES enzyme system such as expressing lytic polysaccharide monooxygenases (LPMO) in the engineered strain cEES, etc.

**Table 4:** Comparison of CNCs preparation methods used in literature and in this study

Treatment methods	Raw material	Characteristics of prepared CNCs	Process overview	References
Sulfuric acid hydrolysis	BEKP	CrI: 73%; length: over 200 nm; Shape: nanofibril-like	Yield 70%; treatment: H_2_SO_4_ 50–64 wt% at 35–80 °C; requiring acid corrosion resistant equipment; great quantity of acid wastewater	Chen et al. (2015)
Sulfuric acid hydrolysis	BEKP	CrI: 66.1–81.9%; width: < 50 nm; length: different size; Shape: rod-like	Highest yield 53.9%; treatment: H_2_SO_4_ 44.1 wt% at 80 °C; requiring acid corrosion resistant equipment, great quantity of acid wastewater	Aguayo et al. (2020)
TEMPO treatment	BSKP	CrI: 81–83%; width: 3.5 nm–3.6 nm; Length: 185 nm–292 nm	Treatment: TEMPO/NaBr/NaClO system in water at pH 10; requiring alkali corrosion resistance equipment; alkaline wastewater containing TEMPO	Zhou et al. (2018)
Cellulase treatment, then sulfuric acid hydrolysis	BSKP	CrI: 75.3%; Particle size distribution: 80 nm–500 nm; Shape: needle-like	Yield 35.3%; treatment: cellulase at 55 °C for 6 h, then H_2_SO_4_ 30–50 wt%; requiring acid corrosion resistant equipment; sugar solution and acid wastewater	An et al. (2016)
Endoglucanase; sulfuric acid	Wood Pulp	CrI: 81–83%; Particle size distribution: 125.2 nm–147.7 nm; Shape: needle	Yield 13.2–16.9%; treatment: cellulase at 50 °C for 72 h and H_2_SO_4_ 64 wt% at 45 °C; requiring acid corrosion resistant equipment; sugar solution and acid wastewater	Dai et al. (2018)
Endoglucanase	BEKP	width: 6 nm–10 nm; length: 400 nm–600 nm	Treatment: cellulase at 50 °C, 72 h; commercia EG-GH5-BA and EG-GH7-TL; sugar solution	Siqueira et al. (2019)
Glycerin; cellulase; xylanase	EP	Size: 30 nm Shape: spherical	Yield 13.6%; optimal reaction conditions: 50 °C, 72 h; commercial cellulase and xylanase; sugar solution	Chen et al. (2018)
cEES cellulase system	EDP	CrI: 87.3–93.4%; Length: < 500 nm Shape: fusiform	Yield 15.7%; reaction conditions: 50 °C, 72 h; engineered cellulase cEES; sugar solution	This study

*BEKP* bleached eucalyptus kraft pulp, *BSKP* bleached softwood kraft pulp, *EP* eucalyptus pulp, *EDP* eucalyptus dissolving pulp, *CrI* crystallinity index

## Conclusion

Using a cellulase system from engineered strain cEES, the CNC was successfully produced from commercial dissolving pulp via enzymatic hydrolysis, and the step-hydrolysis process was more suitable for CNC production than the continuous hydrolysis process. With the progress of enzymatic hydrolysis, the increase in crystallinity of cellulose hinders further enzymatic hydrolysis reactions, but a simple mechanical treatment after the first step of enzymatic hydrolysis can promote the enzymatic hydrolysis for increasing the yields of CNC and glucose as well as improving the size uniformity of CNC. In further work, enzymatic hydrolysis conditions will be optimized for increasing CNC yields, and the cellulase is also expected to be recycled for reducing enzyme costs. The sugar produced in the process will be converted into chemicals through fermentation. This preparation process is pollution-free and has low energy consumption compared to the conventional chemical process, and the biodegradable CNCs are expected to be utilized in the fields of papermaking, packaging, optoelectronic devices, nanocomposite materials, and so on. This study testifies to the potential of the engineered cellulase system cEES in application in enzymatic preparation of CNC, as well as provides a reference process for enzymatic production of CNC.

## Data Availability

All data supporting this article’s conclusion are available.
